# Preventing the next pandemic

**DOI:** 10.2471/BLT.21.020521

**Published:** 2021-05-01

## Abstract

Promising initiatives designed to predict and prevent coming pandemics need to be scaled, connected and coordinated in alignment with a One Health agenda. Lynn Eaton reports.

When the team of researchers arrived in the villages of Boabeng and Fiema wanting to test the monkeys for viruses, Chief Nana Owusu Ameyaw III was somewhat taken aback. “They were concerned that the monkeys might harbour viruses that could be harmful to humans,” he says. “But we are in close daily contact with them and noone has ever become sick.”

Located in the semi-deciduous forest of the Nkoranza District in western Ghana, the monkeys are protected according to local traditions and given the kind of consideration normally reserved for humans.

“We treat them with respect,” the chief explains. “That includes feeding them and making sure they receive a proper burial when they die.”

It was those practices, as well as frequent visits from tourists who come to see and interact with the monkeys, that had brought the team to the village.

Funded by the United States Agency for International Development (USAID), the team was part of the PREDICT project, one of several supported by the agency through its Global Health Security Program. The team was led by Dr Richard Suu-Ire, a senior lecturer at the University of Ghana’s school of veterinary medicine.

Suu-Ire and his colleagues collected samples from the monkeys and the human population as well as from local bats and rodents. The tests on the monkeys and humans came up negative, but Suu-Ire was not disappointed.

“The monkey sanctuary had been identified as a potential transmission hotspot,” he says. “The fact that we didn’t find anything of concern in the human population or in the monkeys was a good outcome as a far as I was concerned. We try to catch outbreaks before they happen.”

For Dr William Karesh, Executive Vice President for Health and Policy at the EcoHealth Alliance, this prospective approach exemplifies modern outbreak surveillance. “The traditional way of assessing the risk of outbreaks is to look at previous occurrences and put dots on a map and say, ‘these are the hotspots’. These days we are looking for risk factors that serve as predictors of future outbreak events and targeting prevention strategies in the places where those factors are occurring,” he says.

One of those risk factors is frequency of contact with wildlife. “It’s been estimated that around 75% of all emerging infectious diseases in humans have some connection to wildlife at some point in time,” Karesh says.

Those diseases include Ebola virus disease, avian and H1N1 influenzas, severe acute respiratory syndrome (SARS) and Middle East respiratory syndrome (MERS) and HIV/AIDS. While the precise origins of the novel coronavirus (SARS-CoV-2) have yet to be established, it may also have made the jump from a wild animal into the human population.

“We need a truly global surveillance system.”

Tracey Goldstein

USAID’s Global Health Security Program, which was launched in 2005, has for the most part focused on potential hotspots in Central and West Africa and in Asia. “We target families of viruses we think have high potential for spillover,” says Dr Tracey Goldstein, who heads up USAID’s Global Health Security Program and previously ran their PREDICT Project.

According to Goldstein, five viral families are of particular concern: filoviruses, which include the Ebola virus; coronaviruses, including SARS, MERS and coronavirus disease 2019 (COVID-19); flaviviruses, such as the Zika virus; paramyxoviruses, including mumps and measles; and influenza viruses.

Engaging with local institutions and communities has been key to boosting prediction capacity. “You can’t just fly in teams of experts and hope to get the job done, you need local ownership and local trust,” says Goldstein.

In Ghana, USAID partnered with the Accra Veterinary Laboratory and the Noguchi Memorial Institute for Medical Research at the University of Ghana, supporting their efforts to increase capacity. “It has been amazing to see the Accra lab expand their capacity, training and staff capability,” she says. “The lab is now doing the testing for SARS-CoV-2 in human samples.”

In Sierra Leone, USAID supported surveillance capacity-building during the 2014 Ebola outbreak, training people to capture and sample animals safely wearing the appropriate personal protective equipment. “Efforts also went into setting up an effective cold chain to get the samples back to the labs which were also upgraded,” Goldstein says. “Those labs are now able to collect and test samples safely.”

The World Bank also sharpened its focus on zoonotic disease surveillance during the 2014–2016 Ebola outbreak with the launching of its Regional Disease Surveillance Systems Enhancement (REDISSE) programme. “The Ebola outbreak exposed the need for engagement and collaboration to try to avoid similar spillover incidents again, and to respond quickly if an outbreak should happen,” says Dr John Paul Clark, head of the REDISSE team.

The World Bank identified areas in West and Central Africa where it judged the risk of emerging zoonotic diseases to be high and set aside US$ 670 million to cover target country requirements over a two-year period. The amount was subsequently increased to just over US$ 1 billion in 2020 to reflect demand generated by the COVID-19 pandemic. There are now 16 countries in West and Central Africa involved in the programme.

“Within each country, the goal is to provide modern disease surveillance systems; to increase laboratory capacity; and to develop a plan for dealing with an outbreak,” Clark says.

Examples of REDISSE capacity-building initiatives include the training of field epidemiologists and laboratory staff using the United States-backed Field Epidemiology and Laboratory Training Program at the Joseph Ki-Zerbo University, Ouagadougou, Burkina Faso, or at the University of Ghana.

Laboratory capacity has also been increased to help detect diseases, with a network of 15 regional reference laboratories which include both human and animal health laboratories and the Pasteur research institutes of Dakar in Senegal and Abidjan in Côte d'Ivoire.

According to Césaire Damien Ahanhanzo, Projects Management Unit General Coordinator with the West African Health Organization (WAHO), the laboratory network is already proving its worth. “WAHO was able to conduct a lab training on COVID-19 diagnostics in February, before cases had reached West Africa,” he says. “All countries were trained, and diagnostic kits were distributed in the region ensuring comprehensive capacity in the region.”

While such initiatives constitute real-world examples of what can be achieved in zoonotic pathogen surveillance, Goldstein believes that in order to meet the challenges likely to be posed by the next global pandemic, they need to be scaled, connected and coordinated.

“Increased animal–human interactions are the result of development policies across many sectors.”

Catherine Machalaba

“We need a truly global surveillance system,” she says. “To achieve that, countries will have to come together supported by the private sector and international organizations including the World Health Organization (WHO), USAID and the World Bank.”

Goldstein believes that scaling up will require not just mobilization of resources but a readiness on the part of the diverse stakeholders to coordinate and collaborate.

Dr Catherine Machalaba, a senior policy adviser with the EcoHealth Alliance, is of the same mind, but stresses that “joining the stakeholder dots” will have to go beyond health system initiatives to embrace all sectors.

“Increased animal–human interactions are the result of development policies across many sectors and include agricultural and industrial policy that encourages encroachment on natural habitats,” she says. “If you want to address these emergencies before they become emergencies, you need to look at the problem from a multisectoral, One Health perspective and develop effective multisectoral information sharing.”

Because development reflects different imperatives, developing a One Health agenda will not be easy. And not just at country level. “There is commitment but a prioritized workplan needs to be developed on the One Health agenda between different United Nations (UN) agencies working on development,” says Francesco Branca, Director of WHO’s Department of Nutrition and Food safety.

Branca has been tasked with establishing a One Health High-Level Expert Panel comprised of independent experts and supported by the UN Food and Agriculture Organization, the World Organisation for Animal Health, the UN Environment Programme and WHO.

“When we talk to different development agencies, there is a wariness about impacting livelihoods,” Branca says. “They say that unless we have a clear demonstration that activities are dangerous, why should we affect the livelihoods of people?”

The Panel is due to be launched in May and it is to be hoped that they will be able to answer that question. The Panel will also be making recommendations regarding the reduction of risk, including possibly with regard to traditional food markets.

Working in collaboration with the World Bank, WHO has already set up a Global Preparedness Monitoring Board and created a Division of Emergency Preparedness as part of efforts to improve its surveillance capacity. WHO also initiated discussions about the drawing up of a pandemic treaty in March, when Director-General Tedros Adhanom Ghebreyesus stated, “Without an internationally coordinated, all-of-government, all-of-society, One Health approach to pandemic preparedness and response, we remain vulnerable.”

Preparedness will also depend on the continued efforts of dedicated researchers like Richard Suu-Ire, who has spent the past two years testing fruit bats in Ghana in search of Henipaviruses, which include Nipah and Hendra viruses, highly pathogenic paramyxoviruses that are considered to be among the next possible outbreak risks. “Part of me hopes I don’t find them,” Suu-Ire says.

**Figure:**
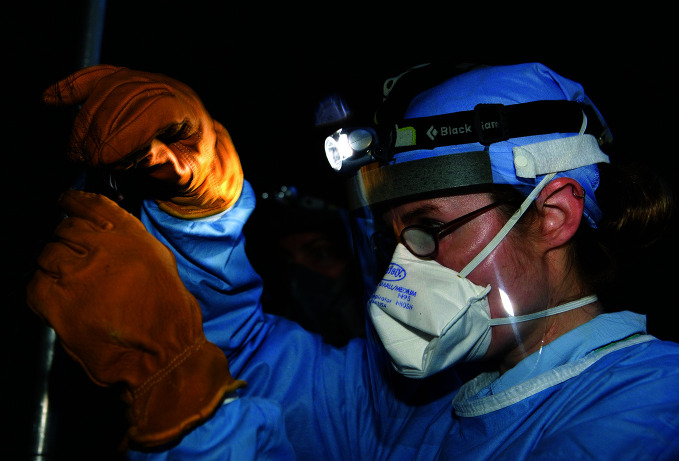
Sampling bats for viruses in Kenya.

WHO/Christopher Black

**Figure:**
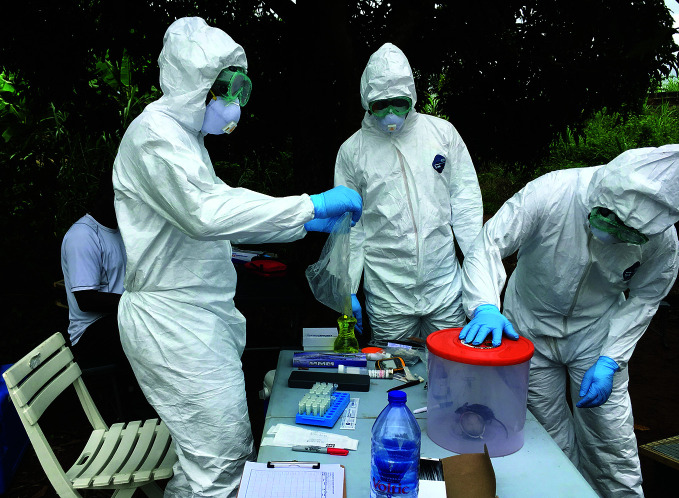
Anaesthetizing rodents prior to sampling in Ghana.

USAID/Richard Suu-Ire.

